# Bis[2-meth­oxy-6-(3-pyridylmethyl­imino­meth­yl)phenolato-κ^2^ *N*,*O*]copper(II)

**DOI:** 10.1107/S1600536809052349

**Published:** 2009-12-12

**Authors:** Xiaodan Chen, Yu Sun, Qingzhao Yao, Huaihong Zhang, Qing Liang

**Affiliations:** a Ordered Matter Science Research Center, College of Chemistry and Chemical Engineering, Southeast University, Nanjing 210096, People’s Republic of China; b College of Pharmacy, Jiangsu University, Zhenjiang 212013, People’s Republic of China

## Abstract

In the title complex, [Cu(C_14_H_13_N_2_O_2_)_2_], the Cu^II^ ion is located on a crystallographic inversion center. The complex thus adopts a square-planar *trans*-[CuN_2_O_2_] coordination geometry, with the Cu^II^ ion coordinated by two 2-meth­oxy-6-(3-pyridylmethyl­imino­meth­yl)phenolate (Schiff base) ligands. The aryl and pyridyl rings in the Schiff base are almost perpendicular to each other, with a dihedral angle of 87.61 (6)° between the planes of the two six-membered rings. The pyridyl ring was refined using a disorder model with approximately 70% occupancy for the major component

## Related literature

For recent developments in functional switching materials, see: Sato *et al.* (2003 ▶). Bis-(*N*-alkysalicylideneimine)copper(II) complexes for induced structural phase transitions, see: Yamada (1999 ▶), and the structural isomers can be isolated, see: Chia *et al.* (1997 ▶). For related metal complexes containing Schiff bases, see: You & Zhu (2004 ▶).
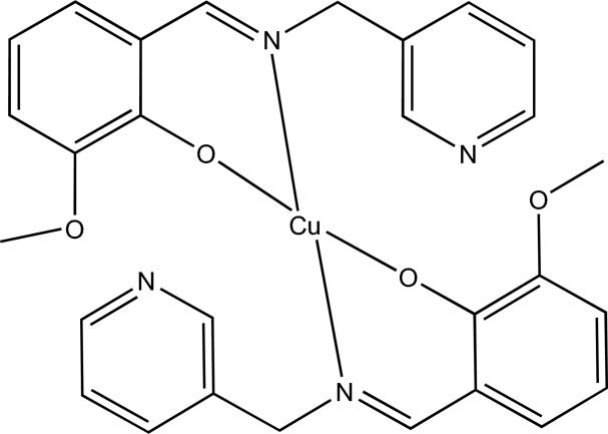


## Experimental

### 

#### Crystal data

[Cu(C_14_H_13_N_2_O_2_)_2_]
*M* *_r_* = 546.07
Triclinic, 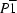

*a* = 5.175 (1) Å
*b* = 10.7291 (14) Å
*c* = 11.4369 (15) Å
α = 99.689 (1)°
β = 91.361 (1)°
γ = 103.241 (2)°
*V* = 608.03 (16) Å^3^
*Z* = 1
Mo *K*α radiation
μ = 0.94 mm^−1^
*T* = 298 K
0.47 × 0.40 × 0.29 mm

#### Data collection

Siemens SMART CCD area-detector diffractometer
Absorption correction: multi-scan (*SADABS*; Sheldrick, 1996 ▶) *T*_min_ = 0.666, *T*_max_ = 0.772
3054 measured reflections
2101 independent reflections
1908 reflections with *I* > 2σ(*I*)
*R*_int_ = 0.021

#### Refinement

*R*[*F* ^2^ > 2σ(*F* ^2^)] = 0.037
*wR*(*F* ^2^) = 0.094
*S* = 1.09
2101 reflections
171 parameters
H-atom parameters constrained
Δρ_max_ = 0.23 e Å^−3^
Δρ_min_ = −0.40 e Å^−3^

### 

Data collection: *SMART* (Siemens, 1996 ▶); cell refinement: *SAINT* (Siemens, 1996 ▶); data reduction: *SAINT*; program(s) used to solve structure: *SHELXTL* (Sheldrick, 2008 ▶); program(s) used to refine structure: *SHELXTL*; molecular graphics: *SHELXTL*; software used to prepare material for publication: *SHELXTL*.

## Supplementary Material

Crystal structure: contains datablocks I, global. DOI: 10.1107/S1600536809052349/nk2015sup1.cif

Structure factors: contains datablocks I. DOI: 10.1107/S1600536809052349/nk2015Isup2.hkl

Additional supplementary materials:  crystallographic information; 3D view; checkCIF report

## Figures and Tables

**Table:** 

Cu1—O1	1.9005 (18)
Cu1—N1	1.997 (2)

**Table:** 

O1—Cu1—N1	91.31 (8)

**Fig. 1.**
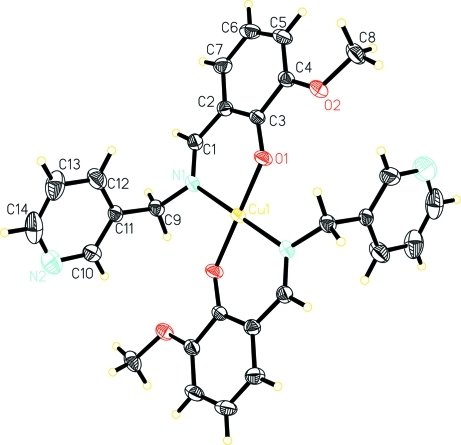
The molecular structure of the title molecule, with the atom-numbering scheme. Displacement ellipsoids are drawn at the 30% probability level.All hydrogen atoms and the minor disorder component are omitted for clarity. [Symmetry code A: -x + 1, -y + 1, -z + 1]

## supplementary crystallographic information

### Comment

Recent developments in functional switching materials (Sato *et al.*, 2003) have shown that a good way to discover candidate metal complexes is to examine compounds that show any changes between two or more states, such as structural phase transitions, isomerism, mixed valences and spin-crossover. In this respect, bis-(*N*-alkysalicylideneimine)copper(II) complexes have potential for induced structural phase transitions (Yamada, 1999) and the structural isomers can be isolated (Chia *et al.*, 1997).

Herein, we reported a new crystal strucure of the title compound (I). The stucture of (I) is shown in Fig.1. It can be clearly seen that this compound possesses a slightly distorted square-planar *trans*-[CuN_2_O_2_] coordination geomentry with the Cu^II^ ion located on a crystallographic inversion center. In addition, the phenyl ring is almost on the same plane with the [CuN_2_O_2_] square plane with a dihedral angle of approximately 6.293° between the two planes, while the pyridyl ring is nearly vertical with the [CuN_2_O_2_] square plane with a dihedral angle of approximately 83.12°. The pyridyl ring was refined using a two-part disorder model, interchanging the positions of N2 and C13 with approximately 70% occupancy for the major component; this conformation is the most likely according to the ring geometry and the surrounding environment.

### Experimental

The title compound was synthesized by Cu(NO_3_)_2_.3H_2_O and Schiff base ligand 2-methoxy-6-[(pyridin-3-ylmethylimino)-methyl]-phenol. All chemicals used (reagent grade) were commercially available. 2-Hydroxy-3-methoxy-benzaldehyde(0.108 g, 1 mmol) was dissolved in ethanol (5 mL) and ethanol solution (5 ml) containing 2-aminoethylpyri dine (0.152 g, 1 mmol) was added slowly with stirring. The resulting yellow solution was continuously stirred for about 30 min. at room temperature, and then Cu(NO_3_)_2_.3H_2_O (0.241 g, 1 mmol) in aqueous solution (5 ml) was added with stirring at room temperature. Brown crystals suitable for X-ray analysis were obtained by slow evaporation at room temperature over several days.

### Refinement

H atoms were placed in geometrical positions and refined using a riding model, with C—H distances in the range 0.93–0.97Å and *U*_iso_(H) =1.5*U*_eq_(C) for methyl groups and *U*_iso_(H) =1.2*U*_eq_(C) for all others. The N2 and C13 atoms in the pyridyl ring was refined using a disorder model with approximately 70% occupancy for the major component

### Crystal data

**Table:**

[Cu(C_14_H_13_N_2_O_2_)_2_]	*Z* = 1
*M**_r_* = 546.07	*F*(000) = 283
Triclinic, *P*1	*D*_x_ = 1.491 Mg m^−^^3^
Hall symbol: -P 1	Mo *K*α radiation, λ = 0.71073 Å
*a* = 5.175 (1) Å	Cell parameters from 13380 reflections
*b* = 10.7291 (14) Å	θ = 3.0–25.0°
*c* = 11.4369 (15) Å	µ = 0.94 mm^−^^1^
α = 99.689 (1)°	*T* = 298 K
β = 91.361 (1)°	Prism, brown
γ = 103.241 (2)°	0.47 × 0.40 × 0.29 mm
*V* = 608.03 (16) Å^3^	

### Data collection

**Table:**

Siemens SMART CCD area-detector diffractometer	2101 independent reflections
Radiation source: sealed tube	1908 reflections with *I* > 2σ(*I*)
graphite	*R*_int_ = 0.021
Detector resolution: 8.192 pixels mm^-1^	θ_max_ = 25.0°, θ_min_ = 1.8°
Thin–slice ω scans	*h* = −5→6
Absorption correction: multi-scan (*SADABS*; Sheldrick, 1996)	*k* = −11→12
*T*_min_ = 0.666, *T*_max_ = 0.772	*l* = −13→13
3054 measured reflections	

### Refinement

**Table:**

Refinement on *F*^2^	Primary atom site location: structure-invariant direct methods
Least-squares matrix: full	Secondary atom site location: difference Fourier map
*R*[*F*^2^ > 2σ(*F*^2^)] = 0.037	Hydrogen site location: inferred from neighbouring sites
*wR*(*F*^2^) = 0.094	H-atom parameters constrained
*S* = 1.09	*w* = 1/[σ^2^(*F*_o_^2^) + (0.0374*P*)^2^ + 0.3354*P*] where *P* = (*F*_o_^2^ + 2*F*_c_^2^)/3
2101 reflections	(Δ/σ)_max_ < 0.001
171 parameters	Δρ_max_ = 0.23 e Å^−^^3^
0 restraints	Δρ_min_ = −0.40 e Å^−^^3^

### Special details

**Table:**

Geometry. All e.s.d.'s (except the e.s.d. in the dihedral angle between two l.s. planes) are estimated using the full covariance matrix. The cell e.s.d.'s are taken into account individually in the estimation of e.s.d.'s in distances, angles and torsion angles; correlations between e.s.d.'s in cell parameters are only used when they are defined by crystal symmetry. An approximate (isotropic) treatment of cell e.s.d.'s is used for estimating e.s.d.'s involving l.s. planes.
Refinement. Refinement of *F*^2^ against ALL reflections. The weighted *R*-factor *wR* and goodness of fit *S* are based on *F*^2^, conventional *R*-factors *R* are based on *F*, with *F* set to zero for negative *F*^2^. The threshold expression of *F*^2^ > σ(*F*^2^) is used only for calculating *R*-factors(gt) *etc*. and is not relevant to the choice of reflections for refinement. *R*-factors based on *F*^2^ are statistically about twice as large as those based on *F*, and *R*- factors based on ALL data will be even larger.

### Fractional atomic coordinates and isotropic or equivalent isotropic displacement parameters (Å^2^)

**Table:**

	*x*	*y*	*z*	*U*_iso_*/*U*_eq_	Occ. (<1)
Cu1	0.5000	0.5000	0.5000	0.03467 (17)	
N1	0.5603 (4)	0.3229 (2)	0.44427 (19)	0.0374 (5)	
C1	0.4647 (5)	0.2520 (3)	0.3428 (2)	0.0396 (6)	
H1	0.5277	0.1775	0.3201	0.048*	
C2	0.2735 (5)	0.2746 (3)	0.2613 (2)	0.0386 (6)	
C3	0.1569 (5)	0.3815 (3)	0.2862 (2)	0.0352 (6)	
C4	−0.0381 (5)	0.3930 (3)	0.2004 (2)	0.0405 (6)	
C5	−0.0989 (6)	0.3047 (3)	0.0952 (3)	0.0501 (8)	
H5	−0.2233	0.3144	0.0395	0.060*	
C6	0.0245 (6)	0.2009 (3)	0.0715 (3)	0.0549 (8)	
H6	−0.0175	0.1423	0.0002	0.066*	
C7	0.2054 (6)	0.1856 (3)	0.1523 (3)	0.0503 (7)	
H7	0.2857	0.1160	0.1362	0.060*	
C8	−0.3148 (6)	0.5261 (4)	0.1431 (3)	0.0615 (9)	
H8A	−0.2139	0.5422	0.0755	0.092*	
H8B	−0.3757	0.6021	0.1751	0.092*	
H8C	−0.4650	0.4540	0.1191	0.092*	
C9	0.7366 (6)	0.2684 (3)	0.5141 (2)	0.0413 (6)	
H9A	0.8854	0.3376	0.5512	0.050*	
H9B	0.8068	0.2052	0.4614	0.050*	
C10	0.7083 (8)	0.2181 (3)	0.7196 (3)	0.0675 (10)	
H10	0.8760	0.2743	0.7354	0.081*	
C11	0.5900 (5)	0.2036 (2)	0.6089 (2)	0.0386 (6)	
C12	0.3393 (7)	0.1225 (4)	0.5890 (3)	0.0689 (10)	
H12	0.2480	0.1087	0.5151	0.083*	
N2	0.6057 (10)	0.1590 (4)	0.8074 (3)	0.0946 (16)	0.70 (4)
C13	0.2220 (8)	0.0611 (4)	0.6786 (5)	0.0910 (17)	0.70 (4)
H13A	0.0530	0.0057	0.6658	0.109*	0.70 (4)
N2'	0.2220 (8)	0.0611 (4)	0.6786 (5)	0.0910 (17)	0.30 (4)
C13'	0.6057 (10)	0.1590 (4)	0.8074 (3)	0.0946 (16)	0.30 (4)
H13B	0.6924	0.1680	0.8736	0.113*	0.30 (4)
C14	0.3650 (12)	0.0834 (5)	0.7849 (4)	0.0863 (14)	
H14	0.2856	0.0419	0.8446	0.104*	
O1	0.2170 (4)	0.47043 (18)	0.38216 (16)	0.0423 (5)	
O2	−0.1501 (4)	0.4964 (2)	0.23188 (18)	0.0514 (5)	

### Atomic displacement parameters (Å^2^)

**Table:**

	*U*^11^	*U*^22^	*U*^33^	*U*^12^	*U*^13^	*U*^23^
Cu1	0.0353 (3)	0.0383 (3)	0.0307 (3)	0.00863 (19)	−0.00441 (18)	0.00810 (19)
N1	0.0374 (12)	0.0430 (13)	0.0360 (12)	0.0136 (10)	0.0011 (9)	0.0134 (10)
C1	0.0449 (16)	0.0396 (15)	0.0368 (15)	0.0137 (12)	0.0045 (12)	0.0083 (12)
C2	0.0395 (15)	0.0416 (15)	0.0320 (14)	0.0029 (12)	0.0028 (11)	0.0080 (11)
C3	0.0314 (14)	0.0420 (15)	0.0303 (14)	0.0018 (11)	−0.0006 (10)	0.0107 (11)
C4	0.0332 (14)	0.0480 (16)	0.0386 (15)	0.0008 (12)	−0.0024 (11)	0.0152 (13)
C5	0.0418 (17)	0.068 (2)	0.0350 (15)	−0.0004 (15)	−0.0078 (12)	0.0134 (14)
C6	0.057 (2)	0.065 (2)	0.0332 (16)	0.0014 (16)	−0.0042 (14)	−0.0019 (14)
C7	0.0575 (19)	0.0477 (17)	0.0406 (16)	0.0074 (14)	0.0005 (14)	0.0011 (13)
C8	0.0455 (18)	0.080 (2)	0.065 (2)	0.0112 (16)	−0.0111 (15)	0.0360 (18)
C9	0.0396 (16)	0.0445 (15)	0.0444 (16)	0.0183 (12)	−0.0024 (12)	0.0100 (12)
C10	0.081 (3)	0.063 (2)	0.048 (2)	−0.0061 (19)	−0.0108 (17)	0.0142 (17)
C11	0.0462 (16)	0.0346 (14)	0.0396 (15)	0.0188 (12)	−0.0011 (12)	0.0075 (11)
C12	0.054 (2)	0.087 (3)	0.067 (2)	0.0038 (19)	−0.0113 (17)	0.036 (2)
N2	0.138 (4)	0.090 (3)	0.049 (2)	0.004 (3)	0.003 (2)	0.0261 (19)
C13	0.063 (3)	0.084 (3)	0.140 (5)	0.015 (2)	0.026 (3)	0.058 (3)
N2'	0.063 (3)	0.084 (3)	0.140 (5)	0.015 (2)	0.026 (3)	0.058 (3)
C13'	0.138 (4)	0.090 (3)	0.049 (2)	0.004 (3)	0.003 (2)	0.0261 (19)
C14	0.125 (4)	0.081 (3)	0.080 (3)	0.052 (3)	0.049 (3)	0.046 (3)
O1	0.0422 (11)	0.0475 (11)	0.0375 (10)	0.0166 (9)	−0.0117 (8)	0.0017 (9)
O2	0.0450 (12)	0.0587 (13)	0.0516 (12)	0.0133 (10)	−0.0159 (9)	0.0141 (10)

### Geometric parameters (Å, °)

**Table:**

Cu1—O1	1.9005 (18)	C8—O2	1.432 (3)
Cu1—O1^i^	1.9005 (18)	C8—H8A	0.9600
Cu1—N1^i^	1.997 (2)	C8—H8B	0.9600
Cu1—N1	1.997 (2)	C8—H8C	0.9600
N1—C1	1.294 (3)	C9—C11	1.512 (4)
N1—C9	1.476 (3)	C9—H9A	0.9700
C1—C2	1.432 (4)	C9—H9B	0.9700
C1—H1	0.9300	C10—N2	1.333 (5)
C2—C3	1.406 (4)	C10—C11	1.362 (4)
C2—C7	1.418 (4)	C10—H10	0.9300
C3—O1	1.306 (3)	C11—C12	1.377 (4)
C3—C4	1.431 (4)	C12—C13	1.389 (5)
C4—O2	1.366 (3)	C12—H12	0.9301
C4—C5	1.381 (4)	N2—C14	1.314 (7)
C5—C6	1.397 (4)	N2—H13B	0.8506
C5—H5	0.9300	C13—C14	1.364 (7)
C6—C7	1.356 (4)	C13—H13A	0.9305
C6—H6	0.9300	C14—H14	0.9300
C7—H7	0.9300		
			
O1—Cu1—O1^i^	180.000 (1)	O2—C8—H8B	109.5
O1—Cu1—N1^i^	88.69 (8)	H8A—C8—H8B	109.5
O1^i^—Cu1—N1^i^	91.31 (8)	O2—C8—H8C	109.5
O1—Cu1—N1	91.31 (8)	H8A—C8—H8C	109.5
O1^i^—Cu1—N1	88.69 (8)	H8B—C8—H8C	109.5
N1^i^—Cu1—N1	180.000 (1)	N1—C9—C11	111.4 (2)
C1—N1—C9	115.3 (2)	N1—C9—H9A	109.3
C1—N1—Cu1	123.32 (19)	C11—C9—H9A	109.3
C9—N1—Cu1	121.18 (18)	N1—C9—H9B	109.3
N1—C1—C2	127.6 (3)	C11—C9—H9B	109.3
N1—C1—H1	116.2	H9A—C9—H9B	108.0
C2—C1—H1	116.2	N2—C10—C11	125.9 (4)
C3—C2—C7	120.3 (3)	N2—C10—H10	117.1
C3—C2—C1	121.9 (2)	C11—C10—H10	117.0
C7—C2—C1	117.8 (3)	C10—C11—C12	115.8 (3)
O1—C3—C2	124.0 (2)	C10—C11—C9	120.7 (3)
O1—C3—C4	118.5 (2)	C12—C11—C9	123.4 (3)
C2—C3—C4	117.5 (2)	C11—C12—C13	120.2 (4)
O2—C4—C5	124.9 (3)	C11—C12—H12	119.9
O2—C4—C3	114.6 (2)	C13—C12—H12	119.8
C5—C4—C3	120.5 (3)	C14—N2—C10	116.2 (4)
C4—C5—C6	120.7 (3)	C14—N2—H13B	121.7
C4—C5—H5	119.6	C10—N2—H13B	122.0
C6—C5—H5	119.6	C14—C13—C12	117.6 (4)
C7—C6—C5	120.1 (3)	C14—C13—H13A	121.5
C7—C6—H6	119.9	C12—C13—H13A	120.9
C5—C6—H6	119.9	N2—C14—C13	124.1 (4)
C6—C7—C2	120.8 (3)	N2—C14—H14	118.6
C6—C7—H7	119.6	C13—C14—H14	117.3
C2—C7—H7	119.6	C3—O1—Cu1	129.26 (17)
O2—C8—H8A	109.5	C4—O2—C8	117.5 (2)
			
O1—Cu1—N1—C1	15.6 (2)	C3—C2—C7—C6	−1.0 (4)
O1^i^—Cu1—N1—C1	−164.4 (2)	C1—C2—C7—C6	179.6 (3)
N1^i^—Cu1—N1—C1	−141 (100)	C1—N1—C9—C11	−99.3 (3)
O1—Cu1—N1—C9	−169.17 (19)	Cu1—N1—C9—C11	85.1 (2)
O1^i^—Cu1—N1—C9	10.83 (19)	N2—C10—C11—C12	1.6 (6)
N1^i^—Cu1—N1—C9	34 (100)	N2—C10—C11—C9	−175.3 (4)
C9—N1—C1—C2	174.5 (2)	N1—C9—C11—C10	−139.2 (3)
Cu1—N1—C1—C2	−9.9 (4)	N1—C9—C11—C12	44.1 (4)
N1—C1—C2—C3	−1.8 (4)	C10—C11—C12—C13	−0.1 (5)
N1—C1—C2—C7	177.6 (3)	C9—C11—C12—C13	176.7 (3)
C7—C2—C3—O1	−177.1 (2)	C11—C10—N2—C14	−2.3 (7)
C1—C2—C3—O1	2.2 (4)	C11—C12—C13—C14	−0.7 (6)
C7—C2—C3—C4	2.5 (4)	C10—N2—C14—C13	1.4 (7)
C1—C2—C3—C4	−178.1 (2)	C12—C13—C14—N2	0.0 (7)
O1—C3—C4—O2	−2.5 (3)	C2—C3—O1—Cu1	10.5 (4)
C2—C3—C4—O2	177.9 (2)	C4—C3—O1—Cu1	−169.14 (17)
O1—C3—C4—C5	177.0 (2)	O1^i^—Cu1—O1—C3	−12 (100)
C2—C3—C4—C5	−2.7 (4)	N1^i^—Cu1—O1—C3	163.5 (2)
O2—C4—C5—C6	−179.3 (3)	N1—Cu1—O1—C3	−16.5 (2)
C3—C4—C5—C6	1.3 (4)	C5—C4—O2—C8	−9.1 (4)
C4—C5—C6—C7	0.3 (4)	C3—C4—O2—C8	170.4 (2)
C5—C6—C7—C2	−0.5 (5)		

Symmetry codes: (i) −*x*+1, −*y*+1, −*z*+1.
